# Exploring Online Health Information–Seeking Behavior Among Young Adults: Scoping Review

**DOI:** 10.2196/70379

**Published:** 2025-09-09

**Authors:** Kristine Stifjell, Torkjel M Sandanger, Charlotte Wien

**Affiliations:** 1 Department of Community Medicine Faculty of Health UiT The Arctic University of Norway Tromsø Norway

**Keywords:** health literacy, health communication, digital health, internet, youth health, SPIDER, PRISMA-ScR

## Abstract

**Background:**

The ability to access and evaluate online health information is essential for young adults to manage their physical and mental well-being. With the growing integration of the internet, mobile technology, and social media, young adults (aged 18-30 years) are increasingly turning to digital platforms for health-related content. Despite this trend, there remains a lack of systematic insights into their specific behaviors, preferences, and needs when seeking health information online.

**Objective:**

This scoping review aims to understand “What previous studies report regarding young adults’ online health information–seeking behavior (OHISB) for health information in terms of the choice of digital platform and platform user interface (UI)?” It attempts to (1) determine which digital platforms young adults tend to use to search for health information and (2) identify characteristics in the UI that apply to young adults’ aims and trust.

**Methods:**

A literature search was conducted in February 2024 across Embase, Web of Science, and Scopus. The final search identified 4634 publications, with 912 publications screened after removing duplicates. Of these, 32 articles met the inclusion criteria. Qualitative content analysis was used to extract themes related to young adults’ OHISB. Studies were selected using predefined eligibility criteria, and data were charted in a structured matrix. Charted data were coded manually and analyzed thematically following the 6-phase framework of Braun and Clarke to identify recurring patterns across the studies. This process was embedded within the Arksey and O’Malley framework for scoping reviews.

**Results:**

The findings showed that young adults primarily use search engines and social media, with information drawn from 5 types of internet-based sources. Six key platform characteristics were found to influence their engagement: credible content, user-friendly design, tailored language, interactive features, privacy, and inclusivity.

**Conclusions:**

Although young adults are active digital health seekers, the current literature does not adequately capture their evolving behaviors. Many studies lacked details on how specific platform affordances influence trust and usability. This review highlights the need for more targeted research on how to design platforms and provides information on how digital context affects online health seeking and decision-making. Given the rapid changes in technologies and information environments, future research should explore their interactions with emerging tools, such as artificial intelligence; address the needs of those in vulnerable situations; and support health literacy in online contexts. An improved understanding of UI preferences and platform behaviors can inform more inclusive, youth-centered digital health interventions.

## Introduction

The rapid evolution of digital technology has revolutionized the way individuals access health information, with the internet becoming a primary resource. This is particularly true for young adults (aged 18-30 years) [1], who are in a critical phase in their lives as they transition to independence and encounter new health challenges [2]. Previous studies on online health information seeking in the general population have identified key influencing factors such as online communities, privacy features [3], quality, and trustworthiness [4]. Young adults’ unique informational needs and their status as a generation inherently familiar with technology from an early age [5] underscore the importance of understanding their online health information–seeking behavior (OHISB). Their approach to finding health information can influence not only their own health outcomes but also those of their peers and family, as they often share what they learn [2,6]. Studying young adults’ OHISB is vital for developing targeted health interventions and resources that cater to their specific needs and habits, facilitating a foundation for future healthy behaviors.

Health information seeking [7] outlines the methods and underlying motivations to actively obtain and use health-related information from various sources [8,9]. The behavior is shaped by personal, demographic, and societal factors such as health literacy [4]. OHISB encompasses the deliberate efforts of individuals to seek out and acquire health-related information from *online platforms* using various digital sources [9]. The term OHISB reflects a range of activities, from the triggers that initiate the search to the strategies employed [10], the types of information sought [4,11], and the outcomes of the search process [3]. This scoping review focuses on the later stages, examining the types of digital platforms young adults choose for health information and the features of user interface (UI) design that influence engagement, trust, and the overall information-seeking behavior [12]. Digital platforms, social media, and artificial intelligence (AI) have expanded the landscape of health information channels, necessitating a comprehensive understanding of how young adults navigate this complex environment to fulfill their health information needs as well as navigate the infodemic [13]. Information is a valuable factor that mediates the relationship between socioeconomic status and health [14]. The evolving dynamics of OHISB could contribute to reducing social inequalities in health [2,4]. From 2010 to 2020, Eurostat reported a 21% rise in online health information seeking among Europeans aged 16-74 years [3]. Further, 52% of individuals reported using the internet for this purpose in 2022 [15]. The engagement exceeded 70% in Nordic countries (Finland, Sweden, Norway, and Denmark), reflecting a significant shift toward digital health literacy [3,16]. This trend is mirrored globally, particularly in Asia, where online health engagement is even more prevalent [3,4]. The digital era has enhanced health autonomy, with young adults valuing the convenience of online resources. This is causing a fundamental shift in the dynamics of patient-provider interactions, with individuals increasingly favoring digital engagement and self-directed care over traditional, in-person consultations. Despite these developments, no prior scoping review has synthesized how platform-specific features influence young adults’ online health information seeking. Given the diversity in platform design and the rapidly evolving digital environment, a systematic overview is needed to identify common patterns, knowledge gaps, and implications for health communication.

The aim of this study is to provide a comprehensive overview of young adults’ OHISB by examining their use of digital platforms and the role of UI design. We conducted a scoping review to (1) explore which platforms young adults tend to use to search for health information and (2) assess the characteristics of the UI that apply to young adults’ aims and trust [17].

This study contributes to the literature by addressing a clear gap in understanding how platform-specific design features affect OHISB in young adults. The findings have potential implications for both practice and theory, informing digital platform developers, health educators, and public health policy makers seeking to improve digital health communication. Theoretically, the study contributes to models of health information behavior by integrating design and engagement factors specific to younger populations.

## Methods

### Overview

The outline for the scoping review was guided by the Arksey and O’Malley framework [18], which was further developed by Levac et al [19,20], and it adhered to the PRISMA-ScR (Preferred Reporting Items for Systematic Reviews and Meta-Analyses extension for Scoping Reviews) guidelines with its 5 stages (identifying the research question; identifying relevant literature; study selection; data charting; and collating, summarizing, and reporting the results) [21]. The research protocol for this scoping review was peer-reviewed and published in BMJ Open [17], outlining the methodological approach, search strategy development, rationale, and anticipated challenges to ensure transparency. The following text includes key components of the protocol while highlighting methodological updates, justifications, and reflections that emerged during the conduct of the review.

### Identifying the Research Question

In this study, OHISB was defined as a deliberate effort to obtain information in response to a recognized need [22] outside general interpersonal communication or media use [9,23,24]. The overall research question for the review was as follows: “What do previous studies report regarding young adults’ OHISB for health information in terms of the choice of digital platform and platform UI?” This can be further presented as two separate aims: (1) determine which digital platforms young adults tend to use to search for health information, and (2) identify the characteristics of the UI that apply to young adults’ aims and trust [17].

### Identifying Relevant Literature

The literature was identified using a search string designed following the SPIDER (Sample, Phenomenon of Interest, Design, Evaluation, Research type) strategy (Table 1) [17,25]. Search terms were derived from Medical Subject Headings (MeSH), keywords, and synonyms identified through Citation Pearl Growing [17,26]. Search terms included a variation of index terms and text words, and Boolean operators (“AND” and “OR”) were used to combine search terms [17,26]. The search string was revisited and developed step by step, and further presented to a group of experts for feedback in order to capture relevant terms related to the overall research question [17].

Comprehensive searches were conducted in 3 electronic databases: Embase, Web of Science, and Scopus. Each database was searched independently to ensure comprehensive coverage and avoid missing relevant studies [17].

**Table 1:** Proposed search strategy mapped against the SPIDER (Sample, Phenomenon of Interest, Design, Evaluation, Research type) tool [17].

Criteria	Search string
Sample	“young adults” OR young OR “early adulthood” OR “early 20s” OR “aged 20” AND
Phenomenon of interest	“Health Information” AND
Evaluation	AND (search* OR seek* OR find* OR access* OR retrieve* OR behavior*) AND (internet OR online OR web OR digital* OR media)

### Study Selection

The time frame for published results was 2017-2024 [9,27,28]. Limiting the scope to the last 5 years ensures relevance to contemporary trends and technologies, capturing the impact of significant recent events like COVID-19 and incorporating emerging platforms like eHealth and social media. The approach also maintains a manageable scope and focuses on up-to-date evidence critically for understanding the current user behavior. Textbox 1 presents the criteria used to include or exclude articles retrieved by the searches. The rationale behind the criteria can be found in the protocol [17].

Based on the criteria, titles, abstracts, and full-text papers were screened manually by 2 reviewers. The results were imported into EndNote and then exported to a screening matrix in Excel (Microsoft Corp).

**Inclusion and exclusion criteria.**

**Inclusion criteria**

- Topic must be related to health
- Study must target young adults (18-30 years old)
- Study must describe online health information seeking behavior activities
- Study must be published in 2017 or later

**Exclusion criteria**

- Study on the use of general information
- Study on communication technology
- Study on behavior change
- Study with a nonspecific age cohort
- Grey literature
- Article published in languages other than English, Norwegian, Swedish, or Danish

### Data Charting, Collating, and Summarizing the Results

The process of charting and synthesizing the data was conducted in accordance with stages 4 and 5 of the Arksey and O’Malley framework, focusing first on the systematic organization of study characteristics and relevant findings, with a subsequent structural analytic synthesis. The review applied structured procedures for data extraction, coding, and thematic analysis to identify patterns across the included studies.

### Data Extraction and Coding Procedure

Data extraction was guided by a structured template developed in line with the review’s objectives and based on the methodological approach outlined in the published protocol [17]. Variables included author, country, sample characteristics, platforms studied, rationale for platform choice, UI features, sender-related indicators, research method, and relevant findings (Multimedia Appendix 1). Charted data were entered into a matrix and coded manually in Excel, using a coding schedule, which was developed by KS and CW. Codes were repeatedly refined during the charting process to ensure they captured patterns relevant to the research question. The coding framework focused on platform use and UI features and provided a foundation for subsequent theme development.

### Thematic Analysis

An inductive thematic analysis was conducted following the 6-phase framework by Barun and Clarke [29]. This included familiarization with the extracted data, generation of initial codes, and identification of repeated patterns related to platform use and UI features. Themes were reviewed and named to reflect the broader meanings across the dataset [29]. The analysis was conducted at a semantic level, emphasizing the explicit content of the included studies. This approach was consistent with the exploratory aim of the scoping review and was conducted within stage 5 of the Arksey and O’Malley framework [17,18], which includes collating, summarizing, and reporting results. Thematic analysis supported the identification and interpretation of common preferences, perceptions, and expectations expressed in the studies by young adults in relation to OHISB.

## Results

### Basic Characteristics of the Included Articles

**Figure 1:**
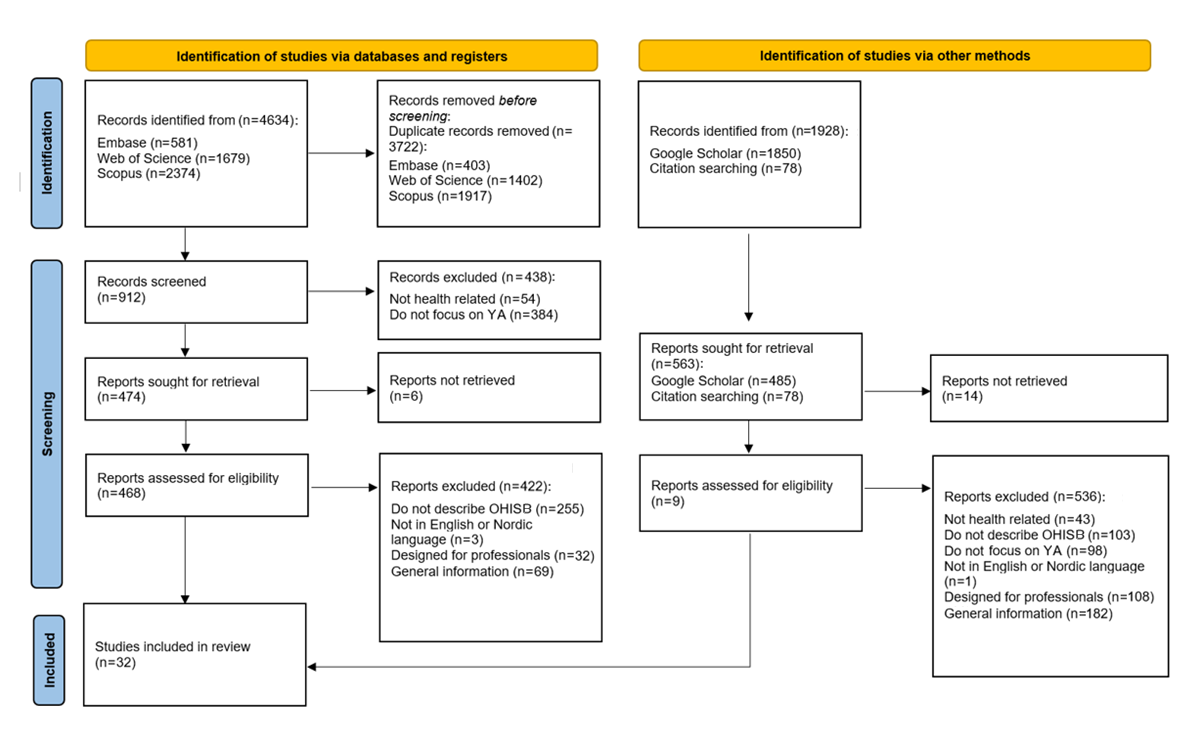
PRISMA (Preferred Reporting Items for Systematic Reviews and Meta-Analyses) 2020 flow diagram. OHISB: online health information–seeking behavior; YA: young adults.

The study selection procedure is displayed in Figure 1 (following the PRISMA-ScR flow diagram to ensure transparency and replicability). After screening, the final sample consisted of 32 articles published between 2017 and 2024 (Multimedia Appendix 2). Aside from a notable peak in 2018, a significant rise in publications on OHISB was noted during the COVID-19 pandemic, where the need for reliable health information became obvious. The shift was driven by not only social distancing but also the possibility of accessing digital resources using the internet and available technology. After the pandemic, the emphasis appears to have shifted toward other pressing research areas. However, the pandemic changed our digital modus operandi. An investigation into the included studies published each year did not reveal any evident trends. However, it is important to determine whether the studies presented overlapping themes, especially regarding trust in health information and platform affordance. The latter will be further investigated.

**Figure 2:**
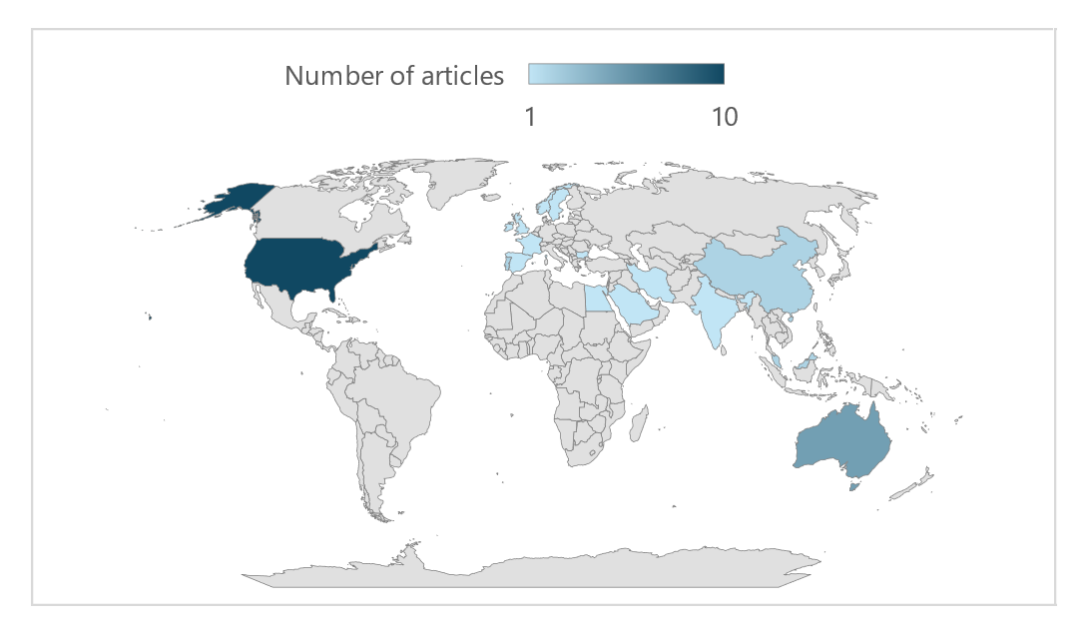
Distribution of publications by country.

All publications were either originally written in English or translated into English. The studies originated from 16 different countries across 5 continents (Figure 2), and this information was determined by the affiliations of the first authors. The distribution was as follows: America (10/32, 31%), Europe (9/32, 28%), Asia (7/32, 22%), Africa (1/32, 3%), and Oceania (5/32, 16%).

The majority of the articles were published in journals. Four journals were frequently cited: Journal of Medical Internet Research (8/32, 25%), Journal of Health Communication (2/32, 5%), Nutrients (2/32, 5%), and Qualitative Health Research (2/32, 5.1%), reflecting the interdisciplinary scope of research on OHISB. Most of the studies were quantitative in nature, representing 63% (20/32) of the included articles, while qualitative research comprised 28% (9/32) of studies, and the remaining 9% (3/32) of studies used mixed methods approaches.

### Demographic Characteristics and Research Methodologies of the Included Articles

The scoping review initially examined how the included articles defined the target population of young adults (Table 2; Multimedia Appendix 3). Of the 32 articles, 15 (47%) described the population using terms like “student” or “college student,” indicating that data collection was focused on cohorts within the age range of 18 to 30 years. The term “young adults” was commonly used alongside “students” in 14 articles. A total of 10 (31%) articles set the upper age cutoff between 29 and 30 years. Moreover, 8 (25%) articles defined young adults as individuals aged 18 to 24 years. Age specification varied, with 6 (19%) studies including individuals between 18 and 30 years and 5 (16%) including individuals between 18 and 25 years. The studies presented a variety of research methods, with 20 (63%) studies implementing quantitative approaches, 9 (28%) implementing qualitative approaches, and 3 (9%) implementing mixed methods approaches.

**Table 2:** Characteristics of the included studies.

Study (first author and publication year)	Title	Journal/conference	Nationality/population country	Sample size	Population (group and age)	Methods
Wang et al [30], 2021	Health information needs of young Chinese people based on an online health community: Topic and statistical analysis	JMIR Medical Informatics	China/China	60,478	Young adults; 18-25 years	Mixed; Data collected from June 1, 2019, to June 1, 2020
Sbaffi et al [31], 2019	Modeling the online health information seeking process: Information channel selection among university students	Journal of the Association for Information Science and Technology	United Kingdom/United Kingdom	291	Students; 19-30 years	Quantitative; Data collected from July 2017
VonHoltz et al [32], 2018	Internet and social media access among youth experiencing homelessness: Mixed-methods study	Journal of Medical Internet Research	United States/United States	87 (survey) + 10 (semistructured interviews)	Homeless youth; 18-21 years	Mixed; Data collected between July 14 and September 12, 2014
Alber et al [33], 2018	Exploring communication strategies for promoting hepatitis B prevention among young Asian American adults	Journal of Health Communication	United States/United States	418	Young Asian American individuals; 18-29 years	Quantitative; Data collection period not available
Molenaar et al [34], 2020	Language of health of young Australian adults: A qualitative exploration of perceptions of health, wellbeing and health promotion via online conversations	Nutrients	Australia/Australia	163	Young adults; 18-24 years	Qualitative; Data collected from May 10 to June 6, 2017
Behre [35], 2022	Young adults’ online sexual health information seeking and evaluating skills: Implications for everyday life information literacy instruction	Proceedings of the Association for Information Science and Technology	United States/United States	65	LGBTQ+^a^ young adults; 18-24 years	Quantitative; Data collected first in November 2020 and then in January 2021. The survey closed in February 2021.
Lee et al [36], 2021	A qualitative analysis of young adults’ health and wellness perceptions, behaviors, and information seeking	Journal of Human Sciences and Extension	United States/United States	34	Young adults; 18-25 years	Qualitative; Data collected from February 2019
Galeshi et al [37], 2018	Influence of ethnicity, gender, and immigration status on millennials’ behavior related to seeking health information: Results from a national survey	Emeral Insights	United States/United States	1082	Millennials; 20-24 years	Qualitative; Data collection: PIAAC 2012/2014 data collected in the United States
Hassan et al [38], 2021	Online health information seeking and health literacy among non-medical college students: Gender differences	Journal of Public Health	Egypt/Egypt	600	University students; 18-30 years	Quantitative; Data collected in March and April 2019
Noorwali et al [39], 2022	Barriers and facilitators to mental health help-seeking among young adults in Saudi Arabia: A qualitative study	Environmental Research and Public Health	Saudi Arabia/Saudi Arabia	12	Young adults; 18-25 years	Qualitative; Data collection period not available
Dix et al [40], 2022	Communicating health to young adults using social media: How, where, and when?	Nutrients	Australia/Australia	2019	Young adults; 18-24 years	Quantitative; Data collection period not available; Protocol published on July 15, 2018
Mendes et al [41], 2017	“That should be left to doctors, that’s what they are there for!”—Exploring the reflexivity and trust of young adults when seeking health information	Health Communication	Portugal/Portugal	15	Young adults; 27 years	Qualitative; Data collected from May 2012 to March 2013
Montagni et al [42], 2018	Exploring digital health use and opinions of university students: Field survey study	JMIR Mhealth Uhealth	France/France	810	University students; 18-24 years	Quantitative, field survey; Data collected from March to April 2017
Ng et al [43], 2018	Factors influencing health information seeking behaviour among young adults in UCSI University	Jurnal Pengajian Media Malaysia	Malaysia/Malaysia	370	Young adults and students; 18-27 years	Quantitative; Data collection period not available
Stankova et al [44], 2020	Health information and CAM online search	Procedia Computer Science	Bulgaria/Bulgaria	731	Young people; 18-30 years	Quantitative; Data collected from July 2018 to April 2020
Pfender et al [45], 2024	An elicitation study to understand young adults’ beliefs about seeking health information from social media influencers	Qualitative Health Research	United States/United States	31	Young adults and undergraduate students; 18-22 years	Data collected in September and October 2022
Zhang et al [28], 2021	Online health information-seeking behaviors and skills of Chinese college students	BMC Public Health	China/China	1203	College students; 18-30 years	Quantitative; Data collected from April to May 2018
Mohamad Shakir et al [46], 2020	Online STI information seeking behaviour and condom use intentions among young Facebook users in Malaysia	Health Promotion International	Malaysia/Malaysia	1530 total; 874 reported online health information–seeking behavior experiences	Young adults; 18-25 years	Quantitative; Data collection: nationwide online survey from December 21, 2015, to February 28, 2016
McCormic et al [47], 2023	Exploring TGE young adults’ experiences seeking health information and healthcare	Youth	United States/United States	42	Transgender or gender expansive; 18-29 years	Quantitative; Data collection period not available
Makesh et al [48], 2020	A study of health information search behaviour and its application among young adults	Indian Journal of Youth and Adolescent Health	India/India	127	Young adults; 18-21 years	Quantitative; Data collected for 7 days in the middle of January 2020
Jalilian et al [49], 2021	Health information seeking behaviors related to COVID-19 among young people: An online survey	International Journal of High Risk Behaviors & Addiction	Iran/Iran	258	Young people; 19-29 years	Quantitative; Data collected from April to May 2020
Pretorius et al [50], 2019	Young people seeking help online for mental health: cross-sectional survey study	JMIR Mental Health	Ireland/Ireland	1308	Young people; 18-25 years	Quantitative; Data collection period not available
Rosário et al [51], 2020	Associations between COVID-19-related digital health literacy and online information-seeking behavior among Portuguese university students	International Journal of Environmental Research and Public Health	Portugal/Portugal	3084	Students; 18-30 years	Quantitative; Data collected from April 28 to June 8, 2020
Porsteinsdóttir et al [52], 2018	Health information seeking among young adults in Sweden	2018 IEEE 31st International Symposium on Computer-Based Medical Systems	Sweden/Sweden	152	Young adults; 18-29 years	Mixed; Data collection period not available
Peñafiel-Saiz et al [53], 2017	Young people, health and the internet. Perceptions, attitudes and motivations of young people in relation to health information	Revista Latina de Comunicacion Social	Spain/Spain	250	Young people; 18-24 years	Quantitative; Data collected from October 2014 to January 2015 and from March to July 2015
Farrugia et al [54], 2021	The “be all and end all”? Young people, online sexual health information, science and skepticism	Qualitative Health Research	Australia/Australia	37	Young people; 18-21 years	Qualitative; Data collection period not available; During the outbreak of COVID-19
Garcia Cosavalente et al [55], 2022	Reproductive health information-seeking: Predictors and perceived barriers among young Peruvian women	World Medical & Health Policy	United States/Peru	635	Young women; 18-26 years	Quantitative; Data collected in December 2019
Waling et al [56], 2022	Embarrassment, shame, and reassurance: emotion and young people’s access to online sexual health information	Sexuality Research and Social Policy	Australia/Australia	37	Young people; 18-21 years	Qualitative; Data collected in 2020
Lim et al [57], 2022	Young adults’ use of different social media platforms for health information: Insights from web-based conversations	Journal of Medical Internet Research	Australia/Australia	165	Young adults; 18-24 years	Qualitative; Data collection began on May 10, 2017, and the website remained active until June 6, 2017
Neely et al [58], 2021	Health information seeking behaviors on social media during the COVID-19 pandemic among American social networking site users: survey study	Journal of Medical Internet Research	United States/United States	1003	Social networking site users; 18 years or older, mean 20.4 years	Quantitative; Data collected from January 9 to January 12, 2021
Khosrowjerdi [59], 2020	National culture and trust in online health information	Journal of Librarianship and Information Science	Norway/United States, China, South Korea	28,371	18-24 years	Quantitative; Data collected from May to June 2017
Kirkpatrick et al [60], 2024	TikTok as a source of health information and misinformation for young women in the United States: Survey study	JMIR Infodemiology	United States/United States	1172	18-29 years	Quantitative; Data collected in April and May 2023

^a^LGBTQ+: lesbian, gay, bisexual, transgender, queer, and others.

### Where do Young Adults Seek Health Information

Sources of information are a commonly discussed element in studies on OHISB [4,61,62]. The majority of articles in the sample represented all web-based sources of health information with the general term “internet” (26/32, 81%) [28,32,33,35-38,41-44,46,47,49-58,60,63,64]. A total of 21 (66%) papers highlighted social media (eg, TikTok and YouTube) as a source of internet-based health information [28,30-35,38-41,47,49,50,52,57-60,63]. Moreover, 13 (41%) publications addressed the use of phones and apps among young adults [32,36,38,40,42,43,49,50,53,54,56,58,63]. Furthermore, 10 (31%) articles identified search engines as the primary online health information resource for young adults, suggesting that young adults still use general search engines (eg, Google) for online health information seeking [28,31,35,38,41,49,51-53,58]. In addition, 9 (28%) studies mentioned government-related health websites (eg, WebMD and CDC) or other websites considered as official and trustworthy [35,40,42,46,49,50,54,60], and 3 (9%) articles mentioned online accessible health portals or platforms [49,51,52].

In summary, young adults typically use 2 main platforms for seeking out health information: search engines and social media. Both allow access to a broad range of online information. When using search engines, the integration of various elements from the obtained articles brings forth 2 distinct categories of information sources and platforms: general internet sources, such as (crowdsourced) encyclopedias, internet forums, or national health websites, and social media. When seeking health information on social media, young adults use platforms they already use for other purposes, such as Instagram, TikTok, YouTube, and WeChat. Their choices are often influenced by the platforms most commonly used in their country of origin.

### Online Platform Characteristics and UI

When young adults choose a platform for seeking out health information, whether a website or social media, several key expectations are evident: (1) credible content from trusted professionals, (2) usability and user-friendly design, (3) tailored language, (4) interaction and web 2.0, (5) privacy, and (6) inclusivity. First, there is a marked preference for platforms supervised by “professionals,” which ensures credibility and the provision of current information [28,31,33,34,41,50,58]. “Professionals” are mainly described as individuals or organizations with verified credentials in health care (eg, licensed practitioners or academic experts). Additionally, there is an expectation for these platforms to actively confront and rectify misinformation [34,58,60]. Second, the esthetic design of these platforms holds considerable significance [32,40,46,51,60,65]. A preference for a “clean” and “straightforward” layout with a UI that is congruent with other familiar digital platforms is evident. With these terms, the literature included examples such as limited text density and consistent visual hierarchy.

Respondents also expressed a wish for a “plastic” UI design that aligns with digital trends in functionality and accessibility [31,32,40]. By plastic, the respondents meant an easy-to-change, evolving, or developing interface that was up to date. Visual aids, such as graphs and short video clips, alongside short segments of written text, were examples [35,46,60,64]. Regarding the third consideration, the language employed on these platforms should be adapted to the health literacy levels of the users, addressing both literacy and genre [30,35,38,51]. It should articulate complex information in a manner that is respectful and not condescending. Short text or a “fact box” is still an effective tool for presenting information that is both accessible and not overly simplistic [28,31]. The fourth consideration involves ensuring that platforms are interactive, aligning with the principles of Web 2.0 (eg, Q&A and social networking service). This includes integrating features that facilitate communication, such as internet forums, comment sections, frequently asked questions [66], and options for direct inquiries to researchers [31]. It is anticipated that these interactive features will be moderated by professionals to uphold the credibility of the platforms [41]. Moreover, the focus on security and anonymity within these platforms is fundamental [28,30,35,39,41,50]. The prevalence of concerns regarding hacking and the potential compromise of personal data are significant [30,47,56]. It is necessary for platforms to prioritize severe security measures to assure user anonymity and raise trust. Lastly, the principle of inclusion is considered essential. This includes a sensitivity to language nuances, the use of personal pronouns, gender identity, and the representation of diversity [35,37,47,56]. Platforms are expected to be inclusive and respectful, mirroring the diverse identities and experiences that characterize the young adult demographic.

## Discussion

### Patterns in Young Adults’ OHISB

The goal of this scoping review was to map the body of literature on young adults’ OHISB regarding the choice of digital platform and the preferences of platform UI. The review offers a unique contribution by synthesizing where young adults seek health information and how platform design influences their trust, engagement, and interpretation of content. Findings from the 32 included studies provide an updated overview on OHISB trends, underscoring that young adults primarily use 2 entry points for digital health information: general search engines and social media. These findings confirm and expand prior reviews by offering a more nuanced understanding of how the affordances of different platforms shape information-seeking behaviors, preferences, and perceived credibility [3,4]. The review applies thematic analysis to group digital sources and interface expectations into coherent patterns, revealing 6 key categories that significantly affect user engagement. These include expectations for credibility, usability, tailored language, interaction, privacy, and inclusivity. A systematic thematic analytical structure regarding young adults` uses and preferences has not been clearly articulated in earlier reviews and contributes an analytical framework for future studies in digital health literacy and communication.

### Social Media Versus Search Engines

Young adults routinely engage with the internet to seek information related to physical and mental health, social identity, and sensitive health topics. Unlike older adults, who typically consult traditional health websites or symptom checkers [67], young adults treat social media platforms as search engines, favoring TikTok, YouTube, and Instagram over Google when seeking answers. This behavioral shift signifies a meaningful change in the OHISB landscape and demands critical attention. The integration of affordances [12], such as comment sections, direct messaging, following, “likes,” video content, and direct messaging, enables young adults to evaluate and interact with health information in ways that go beyond passive consumption. Following the thematic findings, we suggest that the perceived credibility of a message is increasingly tied to parasocial relationships with opinion leaders and content creators [68,69]. These individuals are often viewed as more trustworthy than institutional sources, especially when they use relatable language and shared identities. This trend complicates the role of public health communicators, requiring a rethinking of how trust is established and maintained in digital environments [70].

### Facilitating Online Health Information

**Figure 3:**
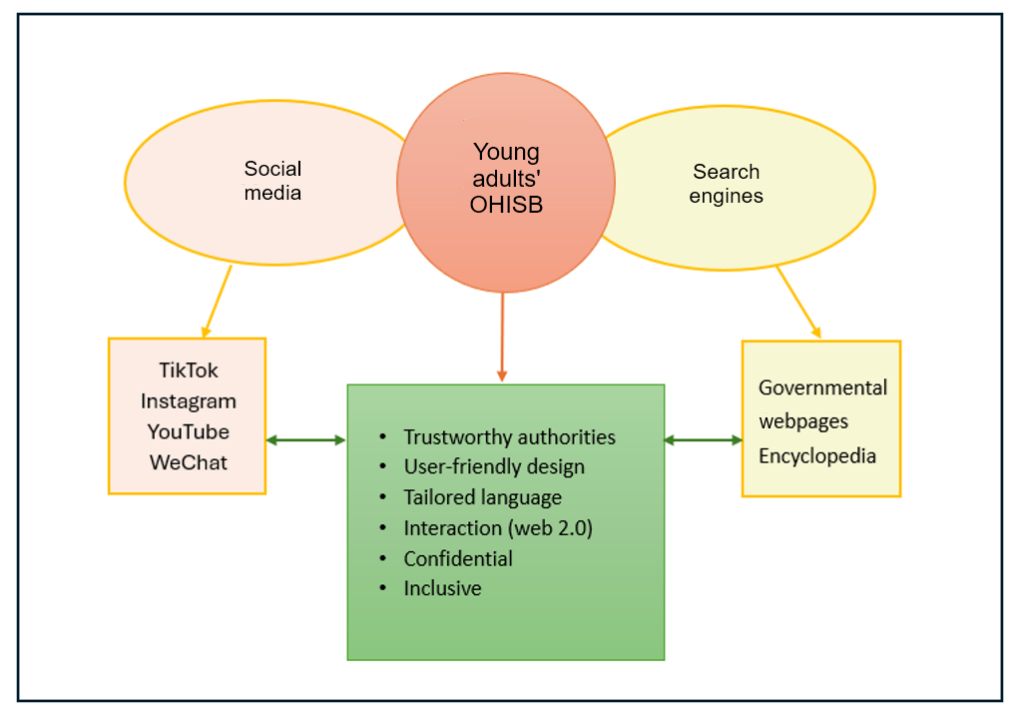
Young adults’ online health information–seeking behavior (OHISB) wish list. Overview of the principal findings.

The findings of this review emphasize that to effectively reach and engage young adults, health information providers must focus on both content and delivery. Figure 3 provides a visual summary of the digital environments young adults most frequently use and highlights their expressed expectations for trustworthy, user-centered, and inclusive digital spaces. To support this population’s health literacy and decision-making, information must be designed with clear language, visual elements, and easy-to-navigate interfaces. Interactivity, anonymity, and representation are also fundamental. These findings align with broader public health goals of equity and access, particularly in a context where misinformation, algorithms, and information overload can hinder informed decision-making. The review suggests that the effectiveness of digital health communication is 2-fold: it is a matter of reaching the right audience and designing content and digital experiences that reflect the preferences and lived realities. By recognizing the platforms that young adults already trust and use and by incorporating UI principles that enhance engagement and credibility, health organizations can better meet the needs of this demographic.

### Expanding the Field of Health Communication and Public Health

This review contributes to the health communication theory by connecting platform affordances with communication outcomes, exemplifying how interface design elements shape trust, literacy, and engagement. The preferences for inclusive design, simplified language, multimodal formats, and real-time interaction align with broader findings in digital health literacy and eHealth behavior change models. The findings inform how public health agencies, nongovernmental organizations, and professionals can design interventions tailored to this age group. Rather than only focusing on the message, effective digital health communication must consider the context of delivery: the platform, the format, and the perceived voice of the communicator. Public health stakeholders must go beyond simply “being present” on social media. Engagement strategies must account for the dynamics of influencer trust, platform-specific language, and the shift toward horizontal rather than top-down communication. This includes ethical considerations around moderation, misinformation management, and inclusion. These findings call for updated frameworks for designing youth-oriented health communication. The thematic clusters identified in this review can serve as design principles for building health interventions across digital ecosystems, especially through interactivity, credibility, and inclusivity. Further, we present suggestions for these design principles by illustrating the key preferences and needs of young adults when seeking health information online (Figure 3).

Figure 3 highlights 2 primary pathways through which young adults access health information: social media platforms, such as TikTok, Instagram, and WeChat (orange box), and more traditional online sources like governmental websites and encyclopedias (yellow box). Across both pathways, young adults consistently express a desire for health information platforms that meet specific criteria (green box). These include trustworthiness, user-friendly design, language tailored to their level of health literacy, interactive figures, data confidentiality, and inclusivity. This visual guide can serve as a practical framework for health communicators and public health professionals seeking to design digital interventions that align with young adults’ expectations and behaviors. Given the fast-changing nature of the digital environment and the increasing reliance on online health resources, these insights underscore the need for more evidence-based approaches to reach and engage this key demographic.

### Conclusions

This scoping review examined how young adults seek health information online, with a focus on the platforms they use and the UI features that influence their engagement, trust, and decision-making. By synthesizing qualitative and quantitative studies from the past 6 years, the review offers a timely and nuanced understanding of the online health information landscape as it relates to younger populations. The thematic synthesis revealed 6 key features that young adults consistently value in digital health platforms: credibility [4,5], user-friendly design, tailored language, interactivity [1,6-8], confidentiality [4,9], and inclusivity [10]. These preferences should not be viewed in isolation, but as interdependent components of a broader user experience that either facilitates or inhibits trust in health information. In addition, the review highlights the evolving nature of platform use, where social media platforms are increasingly functioning as search engines. This shift underscores the urgency for public health institutions to engage directly in the digital spaces young adults inhabit, rather than relying solely on traditional websites or official portals. This review advocates for a more holistic approach to digital health communication: one that integrates user experience design with public health goals and recognizes the complex social and technological ecosystems in which young adults make health decisions. Future research should explore how these principles can be operationalized across diverse contexts and examine how emerging technologies, such as AI-based health tools or immersive media, reshape information-seeking behavior in this population.

### Future Implications

Future research should aim for a more detailed exploration of OHISB, particularly in relation to emerging digital environments, such as social media ecosystems and AI-driven platforms. There is a need for actionable, evidence-based strategies to improve the design and delivery of online health information, with a focus on accessibility, inclusivity, and digital health literacy. Governments, health organizations, and platform developers must actively implement research-based recommendations to create digital environments facilitating access to information and agency. Bridging the gap between research and practice is essential to ensure online health spaces meet the evolving needs of young adults, creating a more accessible, inclusive, secure, and tailored digital health information space.

### Limitations

Research on current trends gets rapidly outdated, and the period of 6 years represents a substantial shift in user behavior. The review aimed to include trends before and after the pandemic, which were marked by rapid changes in people’s internet use and health information consumption. This time lag is a known challenge in research: always a step behind. Creating a search string in collaboration with senior academic librarians, with consideration of existing published filters [3,4], is a notable strength of this review. This approach likely enhanced sensitivity by initially identifying other studies that were not noted in previous reviews focusing on the general population. Another limitation is the number of databases searched (Embase, Scopus, and Web of Science). While the review included 3 broad, interdisciplinary databases that cover a significant portion of the health and social science literature, it did not include PubMed/MEDLINE or PsycINFO. Embase includes all MEDLINE-indexed records, and Scopus partially overlaps with both MEDLINE and psychology journals. PsycINFO remains a unique source for psychological research. Future reviews could benefit from including PsycINFO to ensure discipline-specific studies. Like all literature reviews, there is an inherent risk of compromising validity by excluding relevant papers, possibly due to differences in terminology, the quality of reporting of OHISB, or inconsistent indexing across databases. While the impact of missing papers on the overall interpretation of the findings remains somewhat uncertain, the comprehensive search strategy and targeted scope helped mitigate this limitation.

## Appendix

Multimedia Appendix 1 Data extraction template.

Multimedia Appendix 2 Supporting data.

Multimedia Appendix 3 Detailed characteristics of the included studies.

Multimedia Appendix 4 PRISMA-ScR (Preferred Reporting Items for Systematic Reviews and Meta-Analyses extension for Scoping Reviews) checklist.

## Abbreviations

AI: artificial intelligence
OHISB: online health information–seeking behavior
UI: user interface
