# Supplementary material for: Exploring Online Health Information–Seeking Behavior Among Young Adults: Scoping Review
Source: J Med Internet Res. 2025 Sep 9;27:e70379. doi: 10.2196/70379 (PMC12457860; doi:10.2196/70379)
Supplement: Multimedia Appendix 2 [file jmir_v27i1e70379_app2.docx]

**Multimedia Appendix 2.** Supporting data.

**Table S1.** Distribution of included publication by year.

| **Year** | **Distribution** |
| --- | --- |
| 2017 | 2 |
| 2018 | 6 |
| 2019 | 2 |
| 2020 | 7 |
| 2021 | 6 |
| 2022 | 6 |
| 2023 | 1 |
| 2024 | 2 |
| TOTAL | 32 |

**Table S2.** Where do young adults seek health information

| **Source of online-based health information** | **Number of articles** |
| --- | --- |
| General internet (assorted webpages; Wikipedia, Mayo Clinic) | 26 |
| Social media/blogs (Instagram, TikTok, YouTube, WeChat) | 21 |
| General mobile (e.g. apps, United Healthcare, Ping An Healthcare/Good Doctor) | 13 |
| General search engines (e.g. Google) | 10 |
| Health websites (e.g. WebMD, CDC) | 9 |
| Health portals and platforms | 3 |

**Table S3.** User-interface factors influencing young adults` online health information seeking behavior

| **User-Interface Factors** | **Number of articles** |
| --- | --- |
| Trustworthy authorities | 18 |
| User-friendly design | 13 |
| Interaction (comment-section, direct messages, polls) | 13 |
| Anonymity, confidentiality | 11 |
| Tailored language | 8 |
| Inclusive (language and interface) | 8 |
