# Supplementary material for: Exploring Online Health Information–Seeking Behavior Among Young Adults: Scoping Review
Source: J Med Internet Res. 2025 Sep 9;27:e70379. doi: 10.2196/70379 (PMC12457860; doi:10.2196/70379)
Supplement: Multimedia Appendix 3 [file jmir_v27i1e70379_app3.docx]

**Multimedia Appendix 3.** Detailed characteristics of the included studies.

| **Study** (first author and publication year) | **Title** | **Journal / conference** | **Nationality / population country** | **Sample size** | **Population** (group and age) | **Methods** | **Results** (directly from the studies) |
| --- | --- | --- | --- | --- | --- | --- | --- |
| Wang et al [30], 2021 | Health information needs of young Chinese people based on an online health community: Topic and statistical analysis | JMIR Medical Informatics | China/China | 60,478 | Young adults; 18-25 years | Mixed; Data collected from June 1, 2019, to June 1, 2020 | (…) because of the cultural background and relatively poor sex education level in China, young people are often shy to talk about sexual problems offline, so they hope to receive helpful information online. |
| Sbaffi et al [31], 2019 | Modeling the online health information seeking process: Information channel selection among university students | Journal of the Association for Information Science and Technology | United Kingdom/United Kingdom | 291 | Students; 19-30 years | Quantitative; Data collected from July 2017 | This study investigated the inﬂuence of individual and information characteristics on university students’ information channel selection (ie, search engines, social question and answer sites, online health websites, and social networking sites) of online health information (OHI) for 3 different types of search tasks (factual, exploratory, and personal experience). (...) Search engines are the most frequently used among the 4 channels of information discussed in this study. Credibility, ease of use, style, usefulness, and recommendation are the key factors inﬂuencing users’ judgments of information characteristics (explaining over 62% of the variance). Poisson regression indicated that individuals’ channel experience, age, student status, health status, and triangulation (comparing sources) as well as style, credibility, usefulness, and recommendation are substantive predictors for channel selection of OHI. |
| VonHoltz et al [32], 2018 | Internet and social media access among youth experiencing homelessness: Mixed-methods study | Journal of Medical Internet Research | United States/United States | 87 87 (survey) + 10 ( semi-structured interviews). | Homeless youth; 18-21 years | Mixed; Data collected between July 14 and September 12, 2014 | This study, like ours, identified an interest in reliable resources regarding health and a need among youth experiencing homelessness. In our study, both the survey and qualitative data revealed that the majority of participants used the internet as a primary source of information for health-related matters and that health was very important to those interviewed. Future work in this area can focus on dependable information about common adolescent health issues and about available local resources in a central location. In addition to a resource that focuses on the health needs of youth experiencing homelessness, this study identifies the need and interest in an internet-based platform that addresses the multitude of challenges faced by homeless youth. The results from the qualitative portion of the study further elucidated that limited phone data plans and computer time limits at public libraries had a strong influence on their internet behaviors. As the prevalence of smartphone use is high in this population, any internet outreach efforts must be specifically designed to be compatible with mobile devices. Websites that do not work well on mobile devices may discourage use and reduce the desired outreach effect. This research stresses the importance of digital inclusion, especially with regard to this vulnerable population. Digital inclusion refers to the ability of groups and individuals to gain access to the internet, identify appropriate material and resources, and have opportunities for training to obtain the necessary skills to effectively use available material. |
| Alber et al [33], 2018 | Exploring communication strategies for promoting hepatitis B prevention among young Asian American adults | Journal of Health Communication | United States/United States | 418 | Young Asian American individuals; 18-29 years | Quantitative; Data collection period not available | Results indicated that doctors and health organizations were the most trusted sources of health information, while the internet was the most common source of health information. The majority of participants (99.8%) reported using social media and indicated that they engaged in health-related behaviors on social media. |
| Molenaar et al [34], 2020 | Language of health of young Australian adults: A qualitative exploration of perceptions of health, wellbeing and health promotion via online conversations | Nutrients | Australia/Australia | 163 | Young adults; 18-24 years | Qualitative; Data collected from May 10 to June 6, 2017 | The media, including communications from the government, and social media influencers were seen to perpetuate misconceptions and conflicting messages of what is healthy, which sometimes reduced young adults’ understanding of what a healthy diet looks like. Government messages, such as the Australian Guide to Healthy Eating (AGHE), were often viewed as wrong or hard to believe, as it did not correlate with what they “knew” or other messages online. Some participants who followed an alternative diet to the AGHE, particularly those following a fad diet, did not trust the AGHE messages. To gain exposure from a large audience, it was seen as beneficial to create a “viral” campaign related to fruit and vegetables on social media, with some mentioning the use of a catchy hashtag to help spread content. Going viral was important as it catches people’s attention and creates conversations and a new norm or culture for young adults to engage in. Celebrities, social media influencers, and athletes were suggested as potential spokespeople of the campaign. |
| Behre [35], 2022 | Young adults’ online sexual health information seeking and evaluating skills: Implications for everyday life information literacy instruction | Proceedings of the Association for Information Science and Technology | United States/United States | 65 | LGBTQ+^a^ young adults; 18-24 years | Quantitative; Data collected first in November 2020 and then resent in January 2021. The survey closed in February 2021. | When looking for sexual health information, 89% (n=58) of participants indicated that they prefer searching online. The majority of participants said that they tend to begin their online searches for sexual health information on search engines (n=54, 83%), while others indicated that they turn to the websites of nonprofit organizations like Planned Parenthood (n=5, 8%), medical websites like WebMD or Mayo Clinic (n=4, 6%), social media websites like Twitter (n=1, 2%) or government websites (n=1, 2%). More than two-thirds of the participants (n=44, 68%) reported that they tend not to have any particular website to which they regularly turn for sexual health information; however, 20 participants (31%) did indicate a preferred website. Of those who indicated a preference, Planned Parenthood (n=7, 35%), Mayo Clinic (n=4, 20%), the CDC (n=3, 15%), and Reddit (n=2, 10%) were the most popular choices. (…) When asked why they use the internet to search for sexual health information, many participants indicated that it is easy to access (n=63, 97%), it is easy for them to find the information that they need (n=56, 86%), it is private (n=42, 65%), and it enables them to satisfy their curiosity (n=39; 60%). Four (6%) participants chose to write in additional reasons why they search for sexual health information online. Their responses included feeling ostracized, belonging to a religious and conservative community, having a need to research a specific condition, and wanting to search for others with similar experiences on forums, such as Reddit. It is also relevant to note that 33 (51%) participants indicated that their high school health education did not cover their sexual health information needs. |
| Lee et al [36], 2021 | A qualitative analysis of young adults’ health and wellness perceptions, behaviors, and information seeking | Journal of Human Sciences and Extension | United States/United States | 34 | Young adults; 18-25 years | Qualitative; Data collected from February 2019 | The information-seeking skills and skepticism of information were high for this sample. They were more informed and practiced healthier behaviors related to nutrition and exercise than expected. |
| Galeshi et al [37], 2018 | Influence of ethnicity, gender, and immigration status on millennials’ behavior related to seeking health information: Results from a national survey | Emeral Insights | United States/United States | 1082 | Millennials; 20-24 years | Qualitative; Data collection: PIAAC 2012/2014 data collected in the United States | College-attending young adults tend to rely more on family members and the internet for their questions than their noncollege counterparts (*P*<.05). On the other hand, noncollege young adults tend to rely more on traditional media than their college attending counterparts. |
| Hassan et al [38], 2021 | Online health information seeking and health literacy among non-medical college students: Gender differences | Journal of Public Health | Egypt/Egypt | 600 | University students; 18-30 years | Quantitative; Data collected in March and April 2019 | Students perceived that rapid and easy access to information were the main advantages of health information seeking online (98.5% and 95.5%, respectively), while the lack of reliability and scarcity of information in the Arabic language were the main disadvantages (78.2% and 60.5%, respectively). |
| Noorwali et al [39], 2022 | Barriers and facilitators to mental health help-seeking among young adults in Saudi Arabia: A qualitative study | Environmental Research and Public Health | Saudi Arabia/Saudi Arabia | 12 | Young adults; 18-25 years | Qualitative; Data collection period not available | Online services were also mentioned several times, where many participants agreed that this was a way of facilitating help-seeking while remaining anonymous. Client-therapist confidentiality, more efficient outcomes of online sessions compared to in-person visits, the ability to find a suitable therapist, and more privacy were factors reported by these individuals. However, some participants stated a few concerns regarding online sessions; these included affordability issues and the lack of physical interaction between the therapist and client. |
| Dix et al [40], 2022 | Communicating health to young adults using social media: How, where, and when? | Nutrients | Australia/Australia | 2019 | Young adults; 18-24 years | Quantitative; Data collection period not available; Protocol published on July 15, 2018 | Aspirational healthy eaters are more likely to use smartphones and report high use of Facebook, Instagram, Twitter, Snapchat, Pinterest, and Spotify. (…) Balanced all-rounders are more likely to use smartphones and laptops, and report high use of Facebook, Instagram, and Spotify. (…) Individuals who are designated as contemplating another day are most likely to use a laptop. They report high use of YouTube and Snapchat; share, create, follow, or listen to content; and engage with online entertainment. Blissfully unconcerned individuals are more likely to use smartphones, tablets, and wearable devices, and to report high use of YouTube and Tumblr. They are more likely to follow content online or engage with online entertainment. However, our results indicate that the groups most in need of assistance using social media for health are using it the least. For example, the blissfully unconcerned, in addition to not being concerned about their health, also actively avoid information about health propagated via social media. They do not actively search for information, and they are the least likely to use veracious sources of information overall. The blissfully unconcerned are also less likely to be attracted to content developed by others, even their close social networks. Although only marginally interested (3%) overall, they are interested in commercial content (5.3%) only when such content is provided by their close social network (5.5%). Consequently, affecting the behaviors of the blissfully unconcerned with social media is unlikely and will take more systemic interventions or changes. |
| Mendes et al [41], 2017 | “That should be left to doctors, that’s what they are there for!”—Exploring the reflexivity and trust of young adults when seeking health information | Health Communication | Portugal/Portugal | 15 | Young adults; 27 years | Qualitative; Data collected from May 2012 to March 2013 | The findings show that (1) participants were strongly committed to searching for information about health and lifestyle, especially via the internet; (2) health care professionals were perceived as the most reliable source of health information and advice; and (3) online health information, although frequently accessed and experienced as empowering, was seen as a potentially unreliable source. Findings evidence how becoming better informed about health-related topics plays a pivotal role in individuals’ lives, most notably by using the internet. Participants were able to reflect about what it means to know about health. The construction of trust regarding health information involved a heuristic process vis-à-vis source reliability and perceived credibility that places doctors as the most trustworthy medium of medical advice and health information. We conclude that participants’ trust toward professionals suggests the preference and need for more personalized care, and it is a response to the ambiguity and uncertainty that permeates the health information landscape, particularly that which is web-based. |
| Montagni et al [42], 2018 | Exploring digital health use and opinions of university students: Field survey study | JMIR Mhealth Uhealth | France/France | 810 | University students; 18-24 years | Quantitative, field survey; Data collected from March to April 2017 | Although Wikipedia (357/448, 79.7%) and general health websites (349/448, 77.9%) were the most consulted sources, students considered institutional or official websites as the most credible sources (309/335, 92.2%). There were significant differences in digital health use by gender, field, and year of study. |
| Ng et al [43], 2018 | Factors influencing health information seeking behaviour among young adults in UCSI University | Jurnal Pengajian Media Malaysia | Malaysia/Malaysia | 370 | Young adults and students; 18-27 years | Quantitative; Data collection period not available | The major findings of this study indicate that perceived usefulness was appropriate to act as the main predictor, which empowered young adults to use digital health sources from the web. |
| Stankova et al [44], 2020 | Health information and CAM online search | Procedia Computer Science | Bulgaria/Bulgaria | 731 | Young people; 18-30 years | Quantitative; Data collected from July 2018 to April 2020 | Almost three-quarters of the participants (71%) searched for health-related information on the internet, and they used specialized sites or patient forums. Only 38% reported that they seek information related to alternative and complementary medicine. |
| Pfender et al [45], 2024 | An elicitation study to understand young adults’ beliefs about seeking health information from social media influencers | Qualitative Health Research | United States/United States | 31 | Young adults and undergraduate students; 18-22 years | Data collected in September and October 2022 | Findings from these focus groups demonstrate that youth find many advantages to seeking information from influencers, and it is a behavior widely accepted among their peers, but not necessarily parents and older family members. Barriers to doing so include issues with influencer credibility and trust, but the ease of using social media to find health information seems to outweigh most difficulties. (…) Participants in the study conceptualized an expert not only as someone with professional credentials but also as someone who has embodied experience (Wellman, 2022). (…) This implies that individuals with both formal training and hands-on experience are more likely to garner favorable attitudes from their followers. (…) Young people perceive influencer health information as tailored and relatable, which they cannot find through general Google searches. Social media users also appreciate when influencers test health products and services and provide reviews, which alleviates any effort users spend on trial and error. In this way, participants find that influencer health information is highly credible. |
| Zhang et al [28], 2021 | Online health information-seeking behaviors and skills of Chinese college students | BMC Public Health | China/China | 1203 | College students; 18-30 years | Quantitative; Data collected from April to May 2018 | They sought health information via websites (20.3%), WeChat (2.6%), or both (77.1%). Baidu was the main search engine, and baike.baidu.com (80.3%), Zhihu.com (48.4%), and Zhidao.baidu.com (35.8%) were the top 3 websites among 20 searched websites for information (…). About 20% experienced hacking or internet fraud. |
| Mohamad Shakir et al [46], 2020 | Online STI information seeking behaviour and condom use intentions among young Facebook users in Malaysia | Health Promotion International | Malaysia/Malaysia | 1530 total, 874 reported OHISB experiences | Young adults; 18-25 years | Quantitative; Data collected: nationwide online survey from December 21, 2015, to February 28, 2016 | Among these respondents, most sought sexually transmitted infection (STI) information from online sources (n=874, 91.6%). The majority of respondents sought STI information due to curiosity about STI (n=645, 73.8%). Less than half of the respondents sought STI information because they were worried about STI (n=335, 38.3%) and worried about their sexual health (n=248, 28.4%). A quarter of the respondents wanted to know more about sex (n=222, 25.4%). Having had a partner who had an STI was the least common reason for seeking STI information (n=9, 1.0%). (…) With the increasing number of online users who partake in online dating, it is suggested that STI prevention messages should be advertised on online dating applications, such as Grindr (Huang et al, 2016), as well as on popular virtual chat applications used by teenagers in Malaysia, such as WeChat and BeeTalk, which have been linked to promotion of sexual activity. Furthermore, online sexual health promotion should have messages with well-designed visual aids that will appeal to sexually active young people (Garcia-Retamero and Cokely, 2015). Positively framed messages that describe the benefits of condom use may promote this health protective behavior. |
| McCormic et al [47], 2023 | Exploring TGE young adults’ experiences seeking health information and healthcare | Youth | United States/United States | 42 | Transgender or gender expansive; 18-29 years | Quantitative; Data collection period not available | When participants started to seek information about identities, they started with friends, loved ones, and social media (Twitter, Instagram, and YouTube) (n=5). (…) For many participants, hearing from friends and hearing the personal experiences of others, whether from social media sources or from conversations, were their main sources of information. (…) When asked how they determine if information that they found related to their gender or gender identity was trustworthy, many shared that they were unsure. Examples: “I assumed it was all correct” (Participant #13); “I just hoped to hell it was” (Participant #17); “At the time I didn’t really think about how trustworthy it was. I just assumed it was correct” (Participant #47). At the same time, several discussed “following my instincts” (Participants 6, 10, and 39) or using “what felt right when applied to myself” (Participant #45). Others discussed being able to ask friends in queer health care spaces or asking friends in the community in general and listening to word of mouth. Some found published research related to the topic (Participant #18) or looked at national sites such as Fenway and the University of California at San Francisco (Participant #20). Interestingly, only 1 participant mentioned getting information from their doctor (Participant #9). |
| Makesh et al [48], 2020 | A study of health information search behaviour and its application among young adults | Indian Journal of Youth and Adolescent Health | India/India | 127 | Young adults; 18-21 years | Quantitative; Data collected for 7 days in the middle of January 2020 | Most of the respondents did not use any health-related apps. Apps still have a long way to go as preferred media for health information seeking. (…) The Google Search engine was found to be the most popular way of finding sites related to health, indicating that no one site or platform could be identified as a preferred site for health information. The popularity of social media is another cause for concern, unless they follow known experts. Blogs on health and health-related websites are also accessed online. |
| Jalilian et al [49], 2021 | Health information seeking behaviors related to COVID-19 among young people: An online survey | International Journal of High Risk Behaviors & Addiction | Iran/Iran | 258 | Young people; 19-29 years | Quantitative; Data collected from April to May 2020 | Out of 258 participants, 105 (40.7%) had very good internet search skills, 77 (29.8%) had good skills, and 76 (29.5%) had poor skills. Besides, 194 (75.2%) people started searching for health information from search engines and virtual social media. The highest use of health information sources was related to virtual social media. Also, 243 (94.2%) people reported behavioral improvement after receiving health information. |
| Pretorius et al [50], 2019 | Young people seeking help online for mental health: cross-sectional survey study | JMIR Mental Health | Ireland/Ireland | 1308 | Young people; 18-25 years | Quantitative; Data collection period not available | When looking for help online, 82.57% (1080/1308) of participants made use of an internet search, while 57.03% (746/1308) made use of a health website. When asked about their satisfaction with these resources, 36.94% (399/1080) indicated that they were satisfied or very satisfied with an internet search, while 49.33% (368/746) indicated that they were satisfied or very satisfied with a health website. When asked about credibility, health websites were found to be the most trustworthy, with 39.45% (516/1308) indicating that they found them to be trustworthy or very trustworthy. Most of the respondents (1085/1308, 82.95%) indicated that a health service logo was an important indicator of credibility, as was an endorsement by schools and colleges (719/1308, 54.97%). Important facilitators of online help-seeking included the anonymity and confidentiality offered by the internet, with 80% (1046/1308) of the sample indicating that it influenced their decision a lot or quite a lot. A noted barrier was being uncertain whether information on an online resource was reliable, with 55.96% (732/1308) of the respondents indicating that this influenced their decision a lot or quite a lot. |
| Rosário et al [51], 2020 | Associations between COVID-19-related digital health literacy and online information-seeking behavior among Portuguese university students | International Journal of Environmental Research and Public Health | Portugal/Portugal | 3084 | Students; 18-30 years | Quantitative; Data collected from April 28 to June 8, 2020 | As the pandemic progressed, participants showed a lower chance of achieving a sufficient digital health literacy (DHL) (OR 0.7; 95% CI 0.6-0.9). Using search engines more often (eg, Google) (OR 0.7, 95% CI 0.5-0.9), Wikipedia (OR 0.7, 95% CI 0.6-0.9), and social media (eg, Facebook) (OR 0.7, 95% CI 0.6-0.9) decreased the likelihood of achieving sufficient DHL related to COVID-19. More frequent use of websites of public bodies (OR 1.7, 95% CI 1.1-2.5) increased the odds of reporting sufficient DHL. |
| Porsteinsdóttir et al [52], 2018 | Health information seeking among young adults in Sweden | 2018 IEEE 31st International Symposium on Computer-Based Medical Systems | Sweden/Sweden | 152 | Young adults; 18-29 years | Mixed; Data collection period not available | Respondents mostly report a high regard for the information they retrieve in their searches. The use of support groups for themselves is reported along with an altruistic motivation to support others. While the motivation for many searches is to avoid attending a health professional, approximately one-third search after their visit. |
| Peñafiel-Saiz et al [53], 2017 | Young people, health and the internet. Perceptions, attitudes and motivations of young people in relation to health information | Revista Latina de Comunicacion Social | Spain/Spain | 250 | Young people; 18-24 years | Quantitative; Data collected from October 2014 to January 2015 and from March to July 2015 | Young people use the internet mainly to search for information, to communicate with friends, and to obtain information to do their homework. Although they consult the internet, they prefer to discuss their health problems with their parents or friends. With regards to the roles they play on the internet, they define themselves as trolls, provocateurs, commentators, observers, or creative users. Among the most reliable scientific sources of health information, they rely on books and specialized magazines, displacing newspapers and the internet to secondary positions. |
| Farrugia et al [54], 2021 | The “be all and end all”? Young people, online sexual health information, science and skepticism | Qualitative Health Research | Australia/Australia | 37 | Young people; 18-21 years | Qualitative; Data collection period not available; During the outbreak of COVID-19 | Aside from emphasizing the need for information that addresses young people of diverse genders and sexualities (Grant and Nash, 2019), few of our participants made explicit suggestions for improving online sexual health resources. However, their accounts express a strong desire for resources that are sensitive to the complexity of lived experience. Beyond simply addressing multiple subject positions, such resources could explicitly acknowledge that any information provided is necessarily partial. Crucially, this issue is equally relevant for science-based information as it is for experiential information, both of which, we have argued, generate skepticism. Such an approach would also acknowledge that understandings and experiences of sexual health vary in relation to sociocultural, gender, and sexual identities, among a host of other factors. In this regard, it is noteworthy that many stress the diversity of personal experiences. (…) Most succinctly, our analysis suggests that many young people do not readily trust online sexual health resources. It seems that many desire factual sexual health information produced by experts and backed by credible research. At the same time, however, they express a desire for resources that present lived experiences as well, framing them as sources of insight into phenomena they consider uniquely subjective, such as pleasure and relationships. Importantly, regardless of whether the information sought is seen as factual or experiential, our participants reported a degree of skepticism toward it and described strategies for appraising its credibility. |
| Garcia Cosavalente et al [55], 2022 | Reproductive health information-seeking: Predictors and perceived barriers among young Peruvian women | World Medical & Health Policy | United States/Peru | 635 | Young women; 18-26 years | Quantitative; Data collected in December 2019 | The Comprehensive Model of Information seeking from Johnson and Meischke (1993) was tested. This model indicates that demographics, direct experience, salience, and beliefs predict the utility of information sources. The model also suggests that information-carrier characteristics (perceived trust and perceived utility) could influence health information seeking. Hypotheses related to these variables were tested. An important addition to the model was perceived barriers, which are considered critical factors in the Peruvian context because of the taboo related to sexual and reproductive health. |
| Waling et al [56], 2022 | Embarrassment, shame, and reassurance: emotion and young people’s access to online sexual health information | Sexuality Research and Social Policy | Australia/Australia | 37 | Young people; 18-21 years | Qualitative; Data collected in 2020 | Based on themes emerging from the data, our analysis considers the role of embarrassment and shame in shaping young people’s access to sexual health information, the sources they seek, and the forms of information provision they prefer. Overall, we find that shame, embarrassment, and judgement shape our participants’ access to sexual health information in 4 key ways: by enacting suitable sources of information; by propelling curiosity in different directions; by constituting “normal” bodies, sexuality, and sexual health; and by constituting desired forms of communication. |
| Lim et al [57], 2022 | Young adults’ use of different social media platforms for health information: Insights from web-based conversations | Journal of Medical Internet Research | Australia/Australia | 165 | Young adults; 18-24 years | Qualitative; Data collection began on May 10, 2017, and the website remained active until June 6, 2017 | The factors that young people use to judge credibility and source authority are not consistent with recommendations; these generally suggest using or citing professionals and not testimonial style personal stories (eg, Better Health Channel). Health promoters should attempt to create higher levels of personal trust in our messages, for example, by having real researchers, practitioners, or young adult ambassadors present information directly to young people and building a relationship with their audience. (…) Young adults spent a lot of time scrolling through Facebook newsfeeds, which often resulted in seeing health-related content either from their friends, news sources, or advertisements. Some actively sought out information about specific health areas by joining groups or following relevant pages. YouTube was considered a useful source for learning about everything and was often the go-to when searching for information or advice (after Google). Young adults found the video format easy to learn from. They stated that they could identify accurate YouTube health content by cross-checking multiple videos, by feeling that the presenter was real and relatable, or just through instinctively judging a video’s credibility. Instagram was a source of inspiration for health and wellness from those whose lives were dedicated to healthy lifestyles and fitness. Twitter, Tumblr, and Snapchat were rarely used for health information. |
| Neely et al [58], 2021 | Health information seeking behaviors on social media during the COVID-19 pandemic among American social networking site users: survey study | Journal of Medical Internet Research | United States/United States | 1003 | Social networking site users; 18 years or older, mean 20.4 years | Quantitative; Data collected from January 9 to January 12, 2021 | Our findings highlight the increasing importance of social media in health information seeking and thus highlight its potential value to health professionals as a conduit for personal and public health communications. However, the growing popularity of social networking service (SNS) platforms for health information seeking is not without its potential drawbacks. Among such drawbacks is the noted propensity for SNSs to facilitate the rapid and widespread dissemination of misinformation and disinformation. |
| Khosrowjerdi [59], 2020 | National culture and trust in online health information | Journal of Librarianship and Information Science | Norway/United States, China, South Korea | 28,371 | 18-24 years | Quantitative; Data collected from May to June 2017 | The cultural comparisons revealed differences between the trust formations of Americans, Chinese, and South Koreans in the online health environment. The trust formation of the Chinese group was based on the 3 factors of information quality, information style, and information verification. For the American group, information quality, information style, ease of use, and information verification were the 4 antecedents of trust. |
| Kirkpatrick et al [60], 2024 | TikTok as a source of health information and misinformation for young women in the United States: Survey study | JMIR Infodemiology | United States/United States | 1172 | 18-29 years | Quantitative; Data collected in April and May 2023 | Both health professionals and general users were common sources of health information on TikTok: 93.08% (955/1026) of the participants indicated that they had obtained health information from a health professional, and 93.86% (963/1026) indicated that they had obtained health information from a general user. The respondents showed greater preference for health information from health professionals (vs general users). |

^a^LGBTQ+: lesbian, gay, bisexual, transgender, queer, and others.
