# Supplementary material for: Exploring Online Health Information–Seeking Behavior Among Young Adults: Scoping Review
Source: J Med Internet Res. 2025 Sep 9;27:e70379. doi: 10.2196/70379 (PMC12457860; doi:10.2196/70379)
Supplement: Multimedia Appendix 4 [file jmir_v27i1e70379_app4.docx]

**Multimedia Appendix 4.** PRISMA-ScR (Preferred Reporting Items for Systematic Reviews and Meta-Analyses extension for Scoping Reviews) Checklist.

| **SECTION** | **ITEM** | **PRISMA-ScR CHECKLIST ITEM** | **REPORTED ON PAGE #** |
| --- | --- | --- | --- |
| **TITLE** | | | |
| Title | 1 | Identify the report as a scoping review. | 1 |
| **ABSTRACT** | | | |
| Structured summary | 2 | Provide a structured summary that includes (as applicable): background, objectives, eligibility criteria, sources of evidence, charting methods, results, and conclusions that relate to the review questions and objectives. | 1 – 2 Multimedia Appendix 1: Data Extraction Template Multimedia Appendix 2: Supporting data  Multimedia Appendix 3: Table 2 |
| **INTRODUCTION** | | | |
| Rationale | 3 | Describe the rationale for the review in the context of what is already known. Explain why the review questions/objectives lend themselves to a scoping review approach. | 2 – 3, 4 |
| Objectives | 4 | Provide an explicit statement of the questions and objectives being addressed with reference to their key elements (e.g., population or participants, concepts, and context) or other relevant key elements used to conceptualize the review questions and/or objectives. | 4 – 5  Multimedia Appendix 1: Data Extraction Template |
| **METHODS** | | | |
| Protocol and registration | 5 | Indicate whether a review protocol exists; state if and where it can be accessed (e.g., a Web address); and if available, provide registration information, including the registration number. | 4  A review protocol exists for this scoping review.  It was peer-reviewed and published in *BMJ Open*. The protocol can be accessed here: <https://pmc.ncbi.nlm.nih.gov/articles/PMC10828883/>. |
| Eligibility criteria | 6 | Specify characteristics of the sources of evidence used as eligibility criteria (e.g., years considered, language, and publication status), and provide a rationale. | 5 – 6 Inclusion criteria required studies to focus on health-related topics, target young adults (18–30 years), describe online health information-seeking behavior (OHISB), and be published in 2017 or later. Exclusion criteria included studies on general information use, communication technology, behavior change, non-specific age cohorts, grey literature, and articles published in languages other than English, Norwegian, Swedish, or Danish. The rationale ensures relevance, focus, and contemporary applicability. |
| Information sources* | 7 | Describe all information sources in the search (e.g., databases with dates of coverage and contact with authors to identify additional sources), as well as the date the most recent search was executed. | 5 – 6 The review utilized three databases: Embase, Web of Science, and Scopus. Each database was searched independently to ensure comprehensive coverage. Additionally, we conducted searches on Google Scholar and performed citation tracking using the Pearl Growing method. The most recent search was conducted in February 2024. The search strategy was designed using the SPIDER tool, and terms were refined with feedback from experts to capture relevant studies. |
| Search | 8 | Present the full electronic search strategy for at least 1 database, including any limits used, such that it could be repeated. | EMBASE full search string: ('health information' OR 'health education') AND (search* OR seek* OR find* OR access* OR retriev* OR behavior*) AND ('internet' OR internet OR online OR web OR digital* OR media) AND ('young adult' OR 'young adults' OR young OR 'early adulthood' OR 'early twenties' OR 'aged 20')  Limitations: year 2017 - 2024 |
| Selection of sources of evidence† | 9 | State the process for selecting sources of evidence (i.e., screening and eligibility) included in the scoping review. | 6 – 7  Multimedia Appendix 1: Data Extraction Template  The process for selecting sources of evidence involved a multi-step screening procedure. Titles, abstracts, and full-text articles were manually screened by two independent reviewers based on predefined inclusion and exclusion criteria. Results were imported into EndNote and then exported to a screening matrix in Excel for organization. The PRISMA-ScR flow diagram (Figure 1) was used to ensure transparency and replicability throughout the selection process. Discrepancies between reviewers were resolved through discussion. |
| Data charting process‡ | 10 | Describe the methods of charting data from the included sources of evidence (e.g., calibrated forms or forms that have been tested by the team before their use, and whether data charting was done independently or in duplicate) and any processes for obtaining and confirming data from investigators. | 7 – 8  Multimedia Appendix 1: Data Extraction Template Data charting was guided by a structured template developed in line with the review’s objectives. Variables included author, country, sample characteristics, platforms studied, rationale for platform choice, UI features, sender-related indicators, research method, and relevant findings. Data were entered into a matrix and coded manually in Excel. The coding framework was refined iteratively during the charting process to ensure it captured patterns relevant to the research questions. Discrepancies between reviewers were resolved through discussion. |
| Data items | 11 | List and define all variables for which data were sought and any assumptions and simplifications made. | 7 – 8  Multimedia Appendix 1: Data Extraction Template  The following variables were included in the data extraction process:   - (First) author - Country (conducted + sampled) - Population (age/age range and specific group (e.g., students, young adults). - Sample size - Platforms studied (e.g., search engines, social media) - Rationale for platform use - UI elements - Sender-related indicators (e.g. source, credibility, professional affiliation) - Research method (e.g., quantitative, qualitative, or mixed methods) - Key findings, RQ1 , RQ2 |
| Critical appraisal of individual sources of evidence§ | 12 | If done, provide a rationale for conducting a critical appraisal of included sources of evidence; describe the methods used and how this information was used in any data synthesis (if appropriate). | Critical appraisal was not conducted, as the review aimed to map the breadth of evidence rather than assess study quality. This aligns with JBI methodology and PRISMA-ScR guidelines, which do not require critical appraisal for scoping reviews focused on identifying research gaps. |
| Synthesis of results | 13 | Describe the methods of handling and summarizing the data that were charted. | 7 – 8  Multimedia Appendix 1: Data Extraction Template  Multimedia Appendix 3: Table 2  The charted data were systematically organized into a structured matrix in Excel, focusing on variables such as author, country, sample characteristics, platforms studied, UI features, and relevant findings. The data were coded manually, and the coding framework was refined iteratively to ensure relevance to the research questions. Thematic analysis was then applied to identify recurring patterns and themes across the studies. This process involved grouping codes into broader themes, reviewing and refining these themes, and summarizing them to align with the objectives of the scoping review. The analysis was conducted at a semantic level, emphasizing explicit content in the included studies. |
| **RESULTS** | | | |
| Selection of sources of evidence | 14 | Give numbers of sources of evidence screened, assessed for eligibility, and included in the review, with reasons for exclusions at each stage, ideally using a flow diagram. | 7  Figure 1  The PRISMA flow diagram for the review, which includes searches of databases, registers, and other sources, is presented as Figure 2 (p. 7). During copyediting, the figure was moved to the journal system to comply with journal requirements. Short summary of Fig. 2/ PRISMA flow diagram:  Records identified from SCOPUS, Web of Science (WoS), and EMBASE: 4634  Duplicates removed before screening: 3722  Records screened: 912  Reports assessed for eligibility: 468  Studies identified via other methods: 1928  Reports sought for retrieval: 563  Reports excluded: 536  Reports assessed for eligibility (other methods): 9  Total studies included in the review: 32 |
| Characteristics of sources of evidence | 15 | For each source of evidence, present characteristics for which data were charted and provide the citations. | 10 – 34  Multimedia Appendix 1: Data Extraction Template  Multimedia Appendix 3: Table 2  The characteristics for which data were charted for each source of evidence, along with their citations, are detailed on pages 10–34 in the "Included Articles" section (Table 2).  Charted characteristics:   - Author and Publication Year - Title - Journal. - Nationality/population country - Sample size - Population (group and age) - Methods - Key findings |
| Critical appraisal within sources of evidence | 16 | If done, present data on critical appraisal of included sources of evidence (see item 12). | Critical appraisal of the included sources of evidence was not conducted. |
| Results of individual sources of evidence | 17 | For each included source of evidence, present the relevant data that were charted that relate to the review questions and objectives. | 10 – 34  Multimedia Appendix 1: Data Extraction Template Multimedia Appendix 2: Supporting data  Multimedia Appendix 3: Table 2  The relevant data charted for each included source of evidence are presented in table 2 (p. 10–34). These include details such as author, year, sample characteristics, platforms studied, UI features, and key findings |
| Synthesis of results | 18 | Summarize and/or present the charting results as they relate to the review questions and objectives. | 35 – 37 Multimedia Appendix 2: Supporting data  Multimedia Appendix 3: Table 2  1a) Where YA seek health information:  Internet, web pages, social media, health portals  1b) Platforms YA use for health information:  Search engines, social media, apps  2) Design and UI preferences: Trust, user-friendly design, tailored language, anonymity/security, inclusiveness Detailed document for coding of included studies can be provided upon request. |
| **DISCUSSION** | | | |
| Summary of evidence | 19 | Summarize the main results (including an overview of concepts, themes, and types of evidence available), link to the review questions and objectives, and consider the relevance to key groups. | 35 – 37 Multimedia Appendix 1: Data Extraction Template Multimedia Appendix 2: Supporting data  Multimedia Appendix 3: Table 2  1a) Where: The internet (81.3%) is the primary source, with search engines (e.g., Google) and social media platforms (e.g., TikTok, YouTube, Instagram) being the most used.  2) Platform characteristics: YA prefer platforms with credible content, user-friendly design, tailored language, interactivity, privacy, and inclusivity.  The included studies used quantitative (20 studies = 63%), qualitative (9 studies = 28%), and mixed methods (3 studies = 9%) approaches, covering diverse populations across 17 countries. The results are particularly relevant for public health organizations, platform developers, and policymakers aiming to improve digital health communication for young adults. |
| Limitations | 20 | Discuss the limitations of the scoping review process. | 40  - Time and rapid changes in internet use and health information consumption  - Database coverage: Only three databases  - Terminology and indexing variations  - Exclusion of grey literature |
| Conclusions | 21 | Provide a general interpretation of the results with respect to the review questions and objectives, as well as potential implications and/or next steps. | 37 – 40  Multimedia Appendix 2: Supporting data  Figure 3  The results indicate that young adults primarily use search engines and social media platforms (e.g., TikTok, YouTube, Instagram) for health information, valuing platforms with credible content, user-friendly design, tailored language, interactivity, privacy, and inclusivity. These findings address the review objectives by mapping where young adults seek health information and identifying platform characteristics that influence trust and engagement.  Implications and next steps:   1. Public health organizations and platform developers should design youth-centered digital health interventions that align with young adults' preferences. 2. Future research should explore emerging technologies, such as AI-driven platforms, and their impact on health information-seeking behavior. 3. Efforts should focus on improving accessibility, inclusivity, and digital health literacy to better serve diverse populations. |
| **FUNDING** | | | |
| Funding | 22 | Describe sources of funding for the included sources of evidence, as well as sources of funding for the scoping review. Describe the role of the funders of the scoping review. | 42  The scoping review states that the authors received no financial support for the research or publication of the review. The role of funders is therefore not applicable. |

JBI = Joanna Briggs Institute; PRISMA-ScR = Preferred Reporting Items for Systematic reviews and Meta-Analyses extension for Scoping Reviews.

* Where *sources of evidence* (see second footnote) are compiled from, such as bibliographic databases, social media platforms, and Web sites.

† A more inclusive/heterogeneous term used to account for the different types of evidence or data sources (e.g., quantitative and/or qualitative research, expert opinion, and policy documents) that may be eligible in a scoping review as opposed to only studies. This is not to be confused with *information sources* (see first footnote).

‡ The frameworks by Arksey and O’Malley (6) and Levac and colleagues (7) and the JBI guidance (4, 5) refer to the process of data extraction in a scoping review as data charting*.*

§ The process of systematically examining research evidence to assess its validity, results, and relevance before using it to inform a decision. This term is used for items 12 and 19 instead of "risk of bias" (which is more applicable to systematic reviews of interventions) to include and acknowledge the various sources of evidence that may be used in a scoping review (e.g., quantitative and/or qualitative research, expert opinion, and policy document).

*From:* Tricco AC, Lillie E, Zarin W, O'Brien KK, Colquhoun H, Levac D, et al. PRISMA Extension for Scoping Reviews (PRISMAScR): Checklist and Explanation. Ann Intern Med. 2018;169:467–473. [doi: 10.7326/M18-0850](http://annals.org/aim/fullarticle/2700389/prisma-extension-scoping-reviews-prisma-scr-checklist-explanation).
